# Microbial Transglutaminase as a Tool to Improve the Features of Hydrocolloid-Based Bioplastics

**DOI:** 10.3390/ijms21103656

**Published:** 2020-05-22

**Authors:** C. Valeria L. Giosafatto, Antonio Fusco, Asmaa Al-Asmar, Loredana Mariniello

**Affiliations:** 1 Department of Chemical Sciences, University of Naples “Federico II”, 80126 Naples, Italy; giosafat@unina.it (C.V.L.G.); asmaa.alasmar@unina.it (A.A.-A.); 2 Unità Operativa Struttura Complessa Medicina di Laboratorio, Presidio Ospedaliero Santa Maria di Loreto Nuovo, ASL Na1 Centro, 80145 Naples, Italy; fusco57@virgilio.it; 3 Analysis, Poison control and Calibration Center (APCC), An-Najah National University, P.O. Box 7 Nablus, Palestine

**Keywords:** bioplastics, edible films, technological properties, plastic pollution, enzymatic strengthening

## Abstract

Several proteins from animal and plant origin act as microbial transglutaminase substrate, a crosslinking enzyme capable of introducing isopeptide bonds into proteins between the aminoacids glutamines and lysines. This feature has been widely exploited to modify the biological properties of many proteins, such as emulsifying, gelling, viscosity, and foaming. Besides, microbial transglutaminase has been used to prepare bioplastics that, because made of renewable molecules, are able to replace the high polluting plastics of petrochemical origin. In fact, most of the time, it has been shown that the microbial enzyme strengthens the matrix of protein-based bioplastics, thus, influencing the technological characteristics of the derived materials. In this review, an overview of the ability of many proteins to behave as good substrates of the enzyme and their ability to give rise to bioplastics with improved properties is presented. Different applications of this enzyme confirm its important role as an additive to recover high value-added protein containing by-products with a double aim (i) to produce environmentally friendly materials and (ii) to find alternative uses of wastes as renewable, cheap, and non-polluting sources. Both principles are in line with the bio-economy paradigm.

## 1. Plastic Pollution

**Figure 1:**
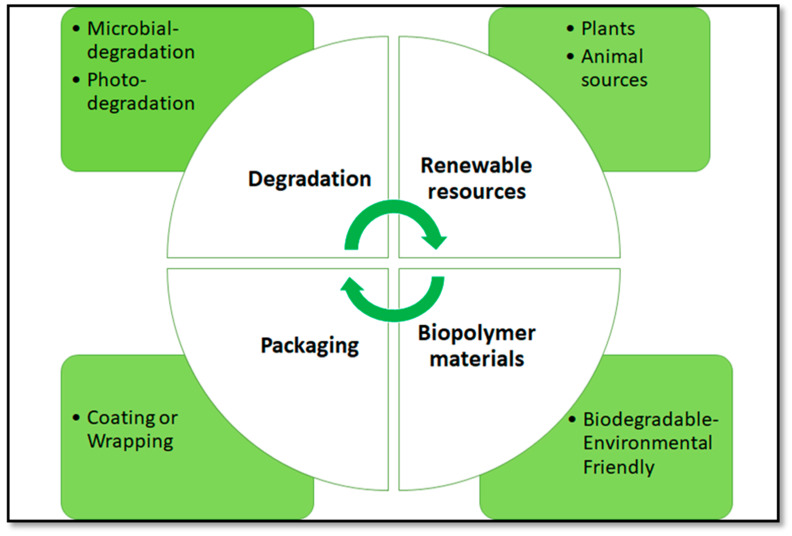
Life cycle of bioplastics (from Popovic et al. [12] with modifications).

Petroleum-based plastics have found widespread applications in our daily life. Their versatility, exceptional features, such as high mechanical resistance, elasticity, and affordability, have been the main reasons for their success [1]. However, although plastics are considered to be one of the greatest innovations in the past century, currently, their wide use is regarded as one of the main causes of environmental pollution as they are not easy to degrade. More than 35 million Tons of wastes from different plastic items are continually produced each year worldwide, and only 7% of them are recycled, the remaining waste being deposited in the landfills or dispersed in the oceans [2,3]. Increased interest in sustainability and consumer trends relating to environmental-friendly products has stimulated researches to find new alternatives to petroleum packaging such as bioplastics produced from renewable sources [4,5,6,7,8]. As a matter of fact, the accumulation of huge amounts of plastic waste in the environment has forced many industrial fields to generate biodegradable plastics. Due to their biodegradability and renewability, bioplastics, by replacing the petroleum-based plastics, can minimize environmental risks. These features are coherent with the main principles of the circular economy, a new area of the economy that, contrary to the linear economy, is based on recycling, reuse, waste management, and eco-efficiency [9]. In fact, bioplastics are made of natural molecules, and after their use, they return to natural molecules [10]. As shown in Figure 1, the application of such materials forms a complete cycle. As a matter of fact, the bioplastics after the degradation process are transformed into renewable resources able to be turned into biopolymer materials that may find application as packaging in the food sector (Figure 1). The bio-based materials, used nowadays especially in the food sector, can have different functions such as food preservation from microbial contamination, extending shelf-life, and controlling the delivery of different molecules like water and gases [11]. In addition, bioplastics can also be used in the pharmaceutical sector as a vehicle of active molecules.

## 2. Protein-Based Bioplastics

### Reinforcement of Bioplastics

**Figure 2:**
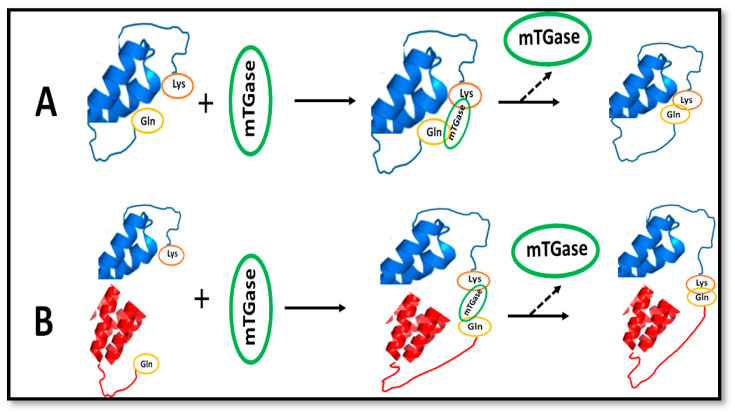
Intra-(**A**) and inter-(**B**) isopeptide bond between glutamine and lysine catalyzed by mTGase (modified from Giosafatto et al. [22]).

Proteins, lipids, and polysaccharides are considered as effective biomolecules for the production of bioplastics. Among polysaccharides, cellulose, chitosan, pectins, and starch are widely used as they can be easily extracted from different agricultural wastes. For example, Giosafatto et al. [13] and Mariniello et al. [14]. exploited the use of pectins from fennel (*Phoeniculum vulgaris*) as biopolymers to obtain hydrocolloid films that might be applied as mulching films. As far as proteins useful for manufacturing bioplastics, different studies exploited both plant proteins such as zein, wheat gluten and soybean [15], and animal-derived proteins, like proteins from milk, collagen, gelatine [16], and egg proteins. The main disadvantages of protein-based, as well as polysaccharide-based films, reside in their low mechanical properties and high sensitivity to moisture that reduce the barrier properties of these materials. In particular, protein-based materials, stable three-dimensional macromolecular networks reinforced by hydrogen bonds, hydrophobic interactions, and disulfide bonds [16], present very low oxygen and carbon dioxide permeability. They should be kept in a dry state since water molecules act as plasticizers, thus, increasing molecular mobility and, therefore, permeability to gases. In addition, since for their nature proteins do not possess sufficient plasticity, a plasticizer is required. Different strategies have been used to solve the disadvantages of protein-based materials with limited success, such as the production of composites and/or the use of nanoparticles and enzymes. In particular, in order to improve the functional properties of hydrocolloid-based films and coatings, the microbial enzyme transglutaminase (mTGase, protein-glutamine γ-glutamyl transferase, E.C. 2.3.2.13), purified from the microorganism *Streptomyces mobaraensis*, has been successfully utilized during the last 18 years [17,18,19]. mTGase is a calcium-independent enzyme able to catalyze the introduction of ε-(γ-glutamyl)-lysine crosslinks into proteins via an acyl transfer reaction. The γ-carboxamide group of glutamine acts as the acyl donor, whereas the ε-amino group of lysine as the acyl acceptor [20], leading to the formation of intra- and inter-molecular crosslinks (Figure 2). In addition, endoprotein reactive lysine may be replaced by several compounds containing primary amino groups, giving rise to a variety of protein (γ-glutamyl) derivatives. In the absence of co-substrates, water can lead to the deamidation of the glutaminyl residue. The biological functions of mTGase remain largely unknown. It has been postulated that *Streptomyces mobaraensis* TGase is able to crosslink inhibitory proteins during the aerial hyphae and spores development [21].

**Figure 3:**
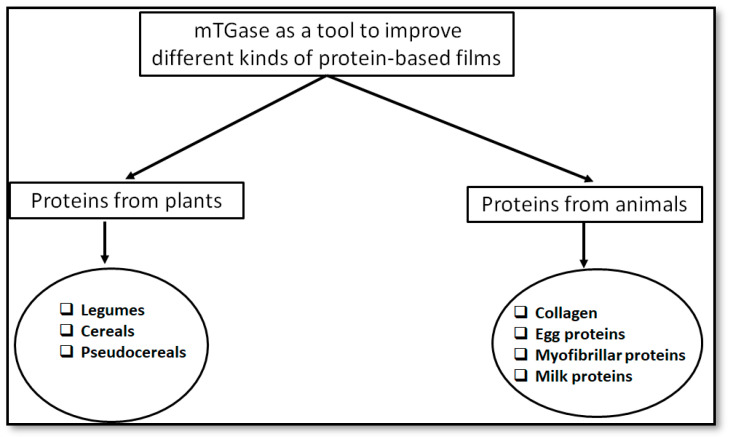
Different source-based proteins able to give rise to bioplastics with improved features following mTGase modification.

In addition, mTGase shows several features, such as activity in a wide range of pH and temperature that are indispensable requisites for an application of the enzyme in the food industry [23]. Furthermore, according to the European legislation (Article 6.4(c) of Directive 2000/13/EC and Article 20 (b) (ii) of Regulation (EU) No 1169/2011), it is not compulsory to declare food enzymes used as processing aids in the list of ingredients on pre-packed food. It is known that most of the proteins of animal and vegetal origins, such as legume globulins, fish proteins, quinoa proteins milk proteins, in addition to many other globulins and albumins, can be efficiently modified by mTGase, giving rise to bioplastics with improved technological properties (Figure 3).

In this regard, several efforts have been carried on to improve the features of bioplastics produced from different protein sources, and some strategies to find applications of the mTGase-derived materials are currently under study and are here discussed. Furthermore, Table 1 summarizes the main features obtained following the microbial enzyme treatment of the different protein-based bioplastics produced in the last two years.

## 3. Proteins From Animals

### 3.1. Collagen

In order to enhance the stability and packing characters of collagen fiber, Wu et al. [28] prepared crosslinked collagen fiber-based edible films using mTGase. Collagen, the most abundant animal protein with a highly ordered structure of repeating units of Gly-X-Y (generally, X and Y are replaced by proline and lysine), is present in skin, bone, blood vessel, and muscle in mammals. Collagen molecules in solution can self-assemble a 4–8 collagen molecule aggregate, forming micro-fibrils via self-aggregation and intermolecular forces. The micro-fibrils have a great stable structure and mechanical resistance [35]. However, a collagen fibril is easily destroyed by temperature rise since its triple helical structure is sensitive to heat, leading to collagen fiber possessing poor thermostability for further application in the packaging sector. According to Cheng et al. [36], mTGase-modified collagen turned into biomaterials with improved features. mTGase crosslinking decreased the thickness of all films, while mechanical properties and thermal stability had a significant improvement, especially at 45 °C and 65 °C. With the proper denaturation temperature and mTGase crosslinking, the tailored film-forming properties of collagen can offer the potential to engineer collagenic material for biodegradable and edible packaging applications. On the other hand, some authors utilized three different thermo-stable proteins of casein, keratin, and soy protein isolate to enhance the thermal stabilities and mechanical properties of collagen fiber films using mTGase crosslinking [37]. The crosslinking greatly reinforced the thermal stability of collagen fiber films, especially that of the collagen fiber crosslinking with 50% casein composite films, as demonstrated by thermogravimetric analysis. For example, for these films, the degradation temperature was higher (of ~26%) than the degradation temperature relative to uncross-linked based-proteins (306 vs. 227 °C, respectively). Furthermore, the mTGase treatment had a positive effect on the mechanical properties of the collagen fiber films in terms of tensile strength and elongation at break. In fact, mTGase-crosslinking on mechanical properties led to a 44.11% increase in tensile strength at dry state and 139.85% at wet state for films prepared with 10% casein, whereas tensile strength reached the largest degree of 60.24% for the heat-treated films made of collagen fiber with crosslinked casein at high concentration (50%). Wu et al. [38] prepared and investigated the features of collagen-based composite films crosslinked by mTGase and containing the antifungal molecule natamycin. The microstructures, functional groups, and physical properties of gelatin films were significantly impacted by the presence of the high concentration of mTGase. Natamycin was successfully incorporated in the mTGase-crosslinked films, and the latter were able to slow down the growth of *Aspergillus ochraceus*, *Aspergillus niger*, and *Penicillium funlculosu*, thus, possessing fungistatic properties. In general, gelatin, the hydrolyzed collagen, comes from skin and cowhide, but for the religious prohibition of Judaism and Islam as well as for health issues about spreading of bovine spongiform encephalopathy since 1986, alternative sources have progressively increased. There are many different options to replace mammalian-based gelatins. Among these, it is worthy of mentioning the gelatin produced from fish skins or bones [39,40]. Yi et al. [41] have treated commercial fish gelatin, commonly found in the skin and bones of cod, haddock, and pollock, with mTGase. They found out that, although the enzyme did not affect the water vapor permeability, it caused an increase in oxygen permeability, tensile strength, melting temperature, and molecular weight. The film color was slightly changed to greenish, and the opacity increased following treatment with mTGase. Alvarado et al. [42] tried to further improve the features of mTGase crosslinked fish-gelatin films by blending the protein with chitosan. The blending was responsible for the improvement of the physical and structural properties of the derived films. 

### 3.2. Myofibrillar Proteins

Myofibrillar proteins, the main component of skeletal muscle, account for about 50% of total proteins. They are mainly constituted by myosin and actin, proteins involved in muscle contraction. Due to their structure and localization [43], myofibrillar proteins are solubilized and extracted under denaturing conditions [44]. There is much research about the functionality of mTGase on these proteins and their effects on the gelation properties. Moreover, it is worthy to point out that myofibrillar proteins from a variety of animal sources edible films are used for edible film preparation, the most utilized ones being those from fish [45] and chicken [46]. Yayli et al. [47] performed some studies on mechanically deboned chicken meat, agricultural by-products containing different proteins. This meat is obtained from edible tissue on chicken bones by deboning or separation techniques. The authors found out that the addition of mTGase to these proteins changed molecular organization and intermolecular interaction in the film matrix. In fact, mTGase-treated films showed a more compact and homogeneous structure than the untreated ones and this, in turn, had a positive influence on film water vapor permeability (that was equal to 1.35 g mm/kPa h m^2^ without mTGase and 2.01 g mm/kPa h m^2^ with mTGase) and mechanical properties. In particular, the tensile strength of films prepared with the microbial enzyme increased by 50%, even though the enzyme decreased the film solubility (from 38% to 32%). On the other hand, Kaewprachua et al. [48] used the myofibrillar proteins from the fish fresh Tilapia to prepare edible wrappings. They demonstrated that the thickness and tensile strength of the films increased as a function of mTGase concentration in the films. In contrast, their extensibility, transparency, water vapor moisture content, and solubility all decreased. However, mTGase was also impacted by ameliorating barrier properties and thermal stability [48]. Other authors have investigated the effect of the incorporation of montmorillonite-based nanoparticles into the film matrix constituted by fish myofibrillar proteins modified by mTGase. They have shown that the inclusion of montmorillonite and mTGase into film enhanced water gain, water vapor permeability, and solubility significantly. It was also shown that the combined effect of clay and mTGase positively affected the mechanical features of nanocomposites [49]. 

### 3.3. Milk Proteins

#### 3.3.1. Caseins

Milk proteins are mostly represented by whey proteins and caseins. Caseins are highly O-glycosylated proteins, whereas whey proteins are mostly N-glycosylated. Briefly, 80% of total milk proteins is represented by caseins that are organized as a large soluble structure, known as micelles. There are three major caseins (α, β, and κ-casein) distributed all over the micelle. The percent of each type of casein varies according to the micelle size, but approximately it is α—55%, β—30%, and κ—15% [50]. Caseins are widely used as additives in different food products such as baked products and cereals, coffee creamers, desserts, and pasta products. Casein derivatives have also been employed in numerous pharmaceutical applications. These casein proteins can be easily modified by both tissue [51] and microbial isoforms [52] of TGase. In particular, Birgitte et al. [51] identified the glutamine and lysine residues involved in guinea pig liver TGase-catalyzed reaction. Chambi and Grosso [52] exploited mTGase to produce casein–gelatin blend edible films. Casein–gelatin films were demonstrated to be more extensible either in the presence or absence mTGase, as compared to films made solely of gelatin or casein. In addition, enzymatic crosslinking led to films with very low permeability. With the microbial enzyme, it was also possible to prepare films in which the molecules of caseins were crosslinked with zein hydrolysate. In such films, wax, either incorporated into the film-forming solution or coated onto the casein films, was exploited. The physical, morphological, and mechanical properties of wax-incorporated or wax-coated casein films were investigated. Wax-casein films showed a darker color than casein films. In addition, wax application significantly reduces both the tensile strength and Young’s modulus of the films [53]. On the other hand, Peng et al. [54] focused on the effect of mTGase treatment on both the physicochemical characteristics and the functional properties of composite films made of succinylated casein/egg white proteins. They found out that the susceptibility of egg white proteins to mTGase-mediated crosslinking improved in the presence of succinylated casein. On the other hand, the films with mTGase appeared more compact with a smoother structure, and the increase in their degree of crystallinity had a positive effect on water resistance, thermal stability, as well as mechanical properties. 

#### 3.3.2. Whey Proteins

Worldwide the dairy industries produce large amounts of a liquid as a by-product following the casein coagulation process. This liquid, known as milk whey (MW), is what remains after recovery of the curd obtained from the acid or proteolytic milk clotting. MW derives from all types (cow, goat, sheep, camel) of milk, even though bovine MW is the prevalent whey produced in the western countries, sharing about 85–95% of the originating milk volume, and containing about 55% of whole milk nutrients [55]. The total worldwide MW production is estimated to be more than 180 million tons/year; the major amount is from the EU and the USA [55]. MW is quite polluting due to its both large volume and high organic content and, thus, its disposal into municipal sewers is practically prohibited everywhere. On the other hand, land dumping creates severe environmental pollution that has a negative effect on soil physicochemical characteristics. Therefore, since MW components are difficult to be degraded, an eco-friendly treatment of MW, when it is not recycled, is required before its discarding, even though the occurrence of numerous nutrients in MW makes it a potential resource for the production of different value-added products [56]. The most preferred method to concentrate whey proteins in their native state is membrane ultrafiltration or nanofiltration, energy-saving technologies producing high-quality products in terms of protein functional properties [57]. MW proteins find numerous applications in the pharmaceutical industry, being therapeutically used to control blood pressure, as well as to induce sleep [58]. Moreover, MW proteins also have the potential to be transformed into bioactive peptides by means of protease or microorganisms. The second procedure involves biotechnological treatments, where MW components are used as substrates for various enzymatic processes to obtain animal feed, probiotics, biopreservatives, and also bioplastics [59]. However, large amounts of MW are not utilized and, thus, MW still deserves attention from researchers to develop further innovative processes able to obtain maximal benefits from this by-product, also to avoid its environmental pollution impact. Di Pierro et al. [60] reported that composite films prepared from mTGase-crosslinked whey proteins may represent a new possible candidate as a substitute for non-edible coating materials for both food and pharmaceutical applications. In particular, they demonstrated that these proteins, following mTGase incubation at a specific pH value called complexation pH (pHc), were able to give rise, in the presence of pectin, to some soluble complexes. The films derived by these supramolecular complexes had an increase of both tensile strength and elongation to break with respect to films prepared in the absence of mTGase. Meanwhile, a significant decrease of elasticity, likely due to the formation of covalent bonds among single whey protein molecules, was observed when the films were prepared in the presence of the enzyme. In addition, the enzyme significantly reduced film permeability. Atomic force and scanning electron microscopy revealed significant changes in the microstructure of the films prepared in the presence of mTGase [60]. Moreover, chitosan–whey protein edible films with different protein concentrations were prepared in the absence or presence of the crosslinking enzyme [61]. The films prepared with the aid of mTGase possessed low solubility at a wide range of pH, a lower degree of swelling, and improved biodegradability following protease treatments. The presence of mTGase also induced an improvement of film mechanical resistance and a reduction in their deformability [61]. Hernàndez-Balada et al. [62], treated whey proteins derived from the cheese industry with mTGase in the presence of gelatin, coming from the leather industry, with the aim to use different protein-containing by-products for the production of bioplastics. They demonstrated the synergistic effect of both proteins following mTGase treatment in improving the functionality of the single biopolymer. 

### 3.4. Egg Proteins

#### 3.4.1. Albumen Proteins

Eggs represent an important source of high-quality proteins and are widely consumed in the Western diet. These, thanks to leavening, emulsification, and binding properties, are important for the preparation of different foods [63]. According to the literature, egg-white contains 24 different glycoproteins, as determined by crossed-immunoelectrophoresis [64]. Lim et al. [65] prepared edible coatings by using egg-white proteins, and, in order to improve tensile and oxygen barrier properties, they proposed a methodology based on preheating treatment under alkaline conditions, followed by incubation of the proteins at 50 °C with the microbial enzyme. Novel composite protein films were also prepared and characterized by Peng et al. [54] that crosslinked egg-white protein and succinylated casein. The prepared films possessed a more homogeneous and smoother structure in the presence of the enzyme and, in turn, possessed better water resistance and thermal stability. The spatial conformation and degree of crystallinity of composite protein film were also affected by mTGase. The increase of crystallinity degree also affected the mechanical properties of the derived films prepared in the presence of mTGase. Among the different egg white proteins, it is worthy to mention ovalbumin since it is the most abundant protein, predominantly contributing to albumen functional properties. It has a molecular weight of 45 kDa and pI of 5.4, consisting of 385 amino acid residues, of which about 50% are hydrophobic. The protein ovalbumin was successfully both intra- and inter-molecularly crosslinked by means of mTGase [66], as shown by the formation of high molecular mass polymers and a monomer with a similar molecular mass but higher electrophoretic mobility when compared to unmodified ovalbumin. Analysis of simulated digestion under physiological conditions has demonstrated that the biopolymers obtained after mTGase treatment were more resistant to both gastric and duodenal digestion. Furthermore, incubation of 1.5% (*w/v*) ovalbumin gel in the presence of mTGase led to the formation of a well-developed viscoelastic gel network with higher modulus and lower phase angle values. Di Pierro et al. [67] made use of mTGase for preparing chitosan/ovalbumin-based composite films. Film swelling was demonstrated to be reduced, whereas the mechanical resistance of the chitosan–ovalbumin films increased following enzymatic treatment. In addition, the permeability toward H_2_O fairly decreased in the films prepared by mTGase-mediated crosslinking.

#### 3.4.2. Yolk Proteins

Marcet et al. [68] prepared edible coatings by using yolk proteins after the extraction of high nutritional value lipids from the yolk. The derived denatured proteins, modified by means of mTGase, gave rise to films that were characterized. Compared to gelatin and caseinate edible films, the films made with egg yolk delipidated protein showed poorer mechanical properties, but improved light barrier properties, low water solubility, and a high degree of transparency. It is particularly interesting that the presence of phosvitin in the egg yolk gives the films important ferrous chelating properties. In addition, when these proteins were treated with the microbial enzyme, the strength of the film was enhanced in comparison with films made with untreated protein. Finally, thymol and natamycin were added to these films to produce shown active biomaterials endowed with antimicrobial properties [68]. 

## 4. Proteins From Plants

### 4.1. Cereal and Pseudocereal Species

#### 4.1.1. Quinoa Proteins

Quinoa (*Chenopodium quinoa*) is an annual herbaceous flowering plant showing interesting nutritional and functional properties due to high-quality protein, particularly rich in lysine. The proteins from mature quinoa seed predominantly are made of 11S-type globulin called chenopodin, comprising of about 37% of the total protein, and also 2S albumin, accounting for 35% of the seed protein [69]. As a consequence, because of the nutritional and functional properties of quinoa proteins, they can be rightly considered as a good candidate to supply human food products. As a matter of fact, there is increasing attention towards quinoa proteins as novel ingredients to improve the nutritional quality of gluten-free bread/pasta [70]. The proteins from quinoa flour were demonstrated to act as both acyl donor and acceptor of mTGase, and it was shown that the derived dough, upon enzyme modification and treatment with proteolytic enzymes, changed the flour microstructures [71]. Moreover, the overall acceptability of the quinoa proteins-containing breads improved in the samples prepared by means of mTGase [71]. As far as the application of quinoa proteins to produce bioplastics, some scientists [27] analyzed the influence of mTGase-crosslinked proteins in two varieties of quinoa. In particular, the films were also prepared by using chitosan, and the physicochemical and barrier properties of the derived composite edible films were evaluated. Crosslinking with mTGase led to a great improvement of edible film properties; nevertheless, interactions among protein and mTGase depended on the quinoa protein variety [27]. In particular, the water vapor permeability of chitosan:quinoa protein ranged from 2.85 to 9.95 × 10^−11^ g cm Pa^−1^ cm^−2^ s^−1^ without mTGase, whereas mTGase reduced the range to 2.42–4.69^11^ g cm Pa^−1^ cm^−2^ s^−1^.

In addition, the films prepared with the enzyme showed also improved thermal stability and a smoother surface as determined by atomic force microscopy. Recently Vera et al. [32] have evaluated the effect of high-intensity ultrasound together with mTGase incubation and the inclusion of nanoparticles on the structural, mechanical, barrier, and physicochemical properties of quinoa protein/chitosan composite edible. It has been shown that the combination of ultrasound and mTGase treatments was responsible for a more pronounced effect on the structure and elongation at break, the latter decreasing of about 2.5 times when the materials were prepared by means of the double treatment [32]. 

#### 4.1.2. Wheat Gluten

Wheat gluten comprises two main groups of water-insoluble and globular proteins represented by gliadins, having low molecular mass, and glutenins with high molecular mass [72]. Edible films were produced from wheat glutens following mTGase modification to improve the physical and barrier properties of the films by using glycerol as a plasticizer. The films prepared from mTGase-modified glutens possessed a lower elongation at break, lower H_2_O permeability, and higher tensile strength than the films prepared with unmodified proteins [73]. mTGase also was able to confer improved water resistance to the protein films [73]. In addition, Larrè et al. [74] produced edible films by using deamidated gluten proteins in the presence of mTGase and of different concentrations of diamines added to the film-forming solutions. The high molecular weight polymers promoted by mTGase between the deamidated gluten and diamines were responsible for a reduction of film solubility as well as of an increase in film integrity and resistance. On the other hand, mTGase-modified gluten proteins edible films characterized by high water vapor permeability and weak mechanical characteristics were prepared by Lai et al. [75]. The authors explained that these properties were likely due to crosslinked bond formation, which created pores and not homogeneous gluten matrix, as evidenced by confocal laser scanning microscopy [75]. Quite recently, Cui et al. [76] exploited the use of α-polylysine to enhance the crosslinking effect of mTGase on gluten, and the authors investigated the properties of the derived gluten films. It was found out that the mTGase-mediated crosslinking increased following the use of α-polylysine. The tensile strength of the films from gluten modified with mTGase and 2% of α-polylysine (g/g gluten) was higher than the films treated with mTGase alone. Moreover, mTGase, together with α-polylysine, was shown to improve the film water stability to a higher extent than the counterpart treated only with the enzyme. mTGase-α-polylysine treated gluten also led to the obtainment of films possessing a rougher surface and a more compact cross-section, as shown by SEM [76].

#### 4.1.3. Corn Zein

Zein is the most important prolamine found in corn endosperm. It is insoluble in water, being dissolved in 70–80% ethanol [77,78]. This protein has been used as a protein component of edible films, and, at different times in the presence of various additives, is able to confer improved performances of the derived films. For example, Cuq et al. [79] and Wang and Padua [80] reported that zein-based films blended with oleic acid improved their water vapor permeability together with flexibility. Barbosa De Almeida et al. [81] and Pena Serna and Lopes Filho [82], reported the enhancement of zein–oleic acid film homogeneity when different concentrations of xanthan gum were added. Although the hydrocolloid molecule was not able to influence film barrier property, xanthan gave rise to films with higher mechanical resistance. Oh et al. [83] developed whey protein or casein films in the presence or absence of mTGase and incorporating zein hydrolysate. The physical and mechanical property characterization of the films demonstrated that zein hydrolysate made whey protein films less soluble, while treatment with the enzyme did not have a significant effect on the solubility. Electrophoresis patterns demonstrated that casein molecules were modified by mTGase, and the extent of the polymerization was further increased when zein hydrolysate was used. In addition, the use of zein hydrolysate decreased the tensile strength of the whey protein films by 35–45%. The elongation of the casein film was increased by 41% because of the action of mTGase and zein hydrolysate, whereas the water vapor permeability of the films did not significantly change. In addition, some authors [84] investigated the effect of four plasticizers (ethylene glycol, propylene glycol, glycerol, and sorbitol) on the mechanical and water barrier properties of mTGase-crosslinked zein–oleic acid films. Results revealed that both mechanical and water barrier properties of the films were greatly influenced by plasticizer type; tensile strength, water vapor permeability, and solubility all improved in ethylene glycol plasticized films compared to other plasticizers. Furthermore, regardless of the plasticizer type, mTGase treatment positively influenced the film tensile strength by 10–15%. The same authors also investigated the best concentration of mTGase able to give rise to zein-based films with the best performances [85] in terms of solubility, mechanical, and barrier properties. They found out that the mechanical and water barrier properties of the films were significantly affected by both pH and drying temperature. Low pH (5 and 6) together with high drying temperature (30 and 35 °C) improved tensile strength, solubility, and water vapor permeability, while high pHs (7 and 8) together with low drying temperature (20 °C and 45% RH, 25 °C) improved elongation at break without enhancing tensile strength, solubility, and water vapor permeability [86]. 

### 4.2. Legumes

#### 4.2.1. Soy Proteins

Soy protein-based products have been demonstrated to be quite nutritious and to deliver health benefits [87]. The two major soy proteins, β-conglycinin (7S) and glycinin (11S), represent 80% of the total proteins in soy. These proteins under heat treatment could be gelled easily by the addition of divalent salts, acid, like glucono-δ-lactone (GDL), and also be extensively enzymatic crosslinked, as demonstrated by Xing et al. [87,88]. As far as bioplastics, one of the first investigations about the use of soy proteins modified by mTGase to prepare edible films dated 2003 when Mariniello et al. [17] first exploited the use of the microbial enzyme to crosslink the soy proteins in the presence of pectins. They found out that pectins were able to promote the formation of high molecular weight protein polymers thanks to electrostatic interactions between polysaccharides and proteins. mTGase-modified soy proteins/pectins composite- based films were successfully obtained, and their mechanical features investigated. The films showed improved tensile strength and elongation at break following mTGase modification of the proteins. A few years later, the same authors [89] also analyzed other properties of these films, e.g., solubility in different pHs and buffers as well as barrier properties towards CO_2_, O_2_, and water vapor. One again, mTGase was able to improve the film features by reducing their solubility and their permeability. In addition, soy proteins were also tested to prepare edible films in combination with a putrescine–pectin conjugate, the latter obtained by means of mTGase. The mTGase-crosslinked films were characterized, and the obtained results demonstrated that a significant decrease of water vapor permeability was observed in the materials obtained with aminated pectins as well as an improvement of mechanical properties [90]. Tang et al. [91] utilized soy proteins isolate following mTGase-mediated crosslinking to prepare biodegradable cast film. They verified that the enzyme was able to influence the structure of the films by making the surface rougher and the cross-section more homogeneous and compact. The presence of mTGase improved film tensile strength and hydrophobicity while reducing elongation at break, moisture content, and transparency. The effect of mTGase treatment on the properties of cast films prepared from soy protein isolate in the presence of sodium caseinate, gelatin, whey protein concentrate, wheat gluten, and peanut protein isolate was also investigated [92]. The results indicate that mTGase significantly improved the tensile strength and surface hydrophobicity of most of the films obtained and resulted in the decrease in the water content, the total soluble matter, and the transparency for all films. Quite recently, Wu et al. [28,38] made some efforts to further improve the collagen-based films in the presence of different proteins, like soy protein isolate, keratin, and casein. They found out that the materials showed enhanced thermal stability [38] and improved barrier and mechanical properties [28], compared with films prepared exclusively with collagen.

#### 4.2.2. White Bean Proteins

White beans (*Phaseolus vulgaris L*.) are widely grown and consumed in developed as well as developing nations of the world, supplying significant amounts of protein, starch, unsaturated fatty acids, dietary fiber, mainly soluble fiber, besides being an excellent source of some minerals (iron and zinc) and vitamins [93]. Hence, representing a good source of proteins in human diets, they are consumed worldwide [94]. The bean proteins act, as the proteins from different legumes, as mTGase substrate [22,93,94]. In particular, Romano et al. [93] investigated the influence of mTGase treatment on several properties of *Phaseolus vulgaris* flour prepared from both whole and shelled beans. Scanning electron microscopy observations of starch and proteins showed an evident effect of mTGase on the structure of flour samples. In fact, when mTGase was added, bean flour macromolecules appeared immersed in a closed, packed structure, which might be attributed to the formation of mTGase-catalysed heteropolymers. Whole beans flour and shelled beans flour presented a darker color and higher water-holding capacity than their mTGase-treated counterparts. mTGase had a significant effect on the thermal parameters of flour samples, which indicated an increase in resistance of the starch within the granules to gelatinization. In addition, mTGase through the formation of ε-(γ-glutamyl) lysine isopeptide bonds affects the gastric digestibility of the bean flour. It is worth to point out that the major storage protein in the cotyledons of the bean *Phaseolus vulgaris* is phaseolin, a globular protein with structural properties very similar to those of the 11S globulins from soybean and other legumes. This globulin was proven to act as mTGase substrate giving rise to intra- and inter-molecular crosslinks [14]. mTGase-modified phaseolin was used as a protein component of composites films prepared in the presence of *Citrus* pectins [4]. These films performed very well in terms of mechanical and barrier properties. In addition, the mTGase-crosslinked phaseolin was also blended with fennel homogenates giving rise to films with good biodegradability properties, making them suitable as mulching films [14] to replace the plastic ones very polluting for the environment. In another study, Mariniello et al. [19] used the protein following enzyme modification to prepare edible films in the presence of grapefruit albedo as a source of polysaccharides. Albedo, the inner part of the peel representing the layer of white spongy material that directly protects the juicy part of the fruit, is quite rich in pectins. Swelling degree, barrier properties to water vapor, oxygen and carbon dioxide, and mechanical performances of such films, were investigated. The addition of the protein, mostly in the presence of mTGase, provides films less swellable compared to films made from albedo homogenates only, whereas the action of the enzyme clearly was demonstrated to provide films that were more stretchable. Moreover, mTGase-mediated crosslinking of phaseolin gives rise to films with enhanced barrier properties toward carbon dioxide and water vapor. These findings make albedo–phaseolin film prepared in the presence of mTGase a promising candidate to be used in food wrapping.

#### 4.2.3. Bitter Vetch Proteins

Bitter vetch, an annual legume cultivated for animal feeding, is endowed with seeds rich in proteins that possess good film-forming properties [95,96]. For this reason, the proteins from this legume are considered as promising biopolymers source to substitute petroleum-derived plastics. To further improve the properties of bitter vetch protein-based materials, the possibility to blend the proteins with polyamines, carbohydrates, nanoparticles [6,7,24,95] in the presence or absence of mTGase was investigated, since these proteins act as effective substrates of the enzyme [6]. In fact, the enzyme was able to reinforce the bitter vetch based-films by making the material structure more compact and smoother comparting to films obtained without mTGase [6]. The effect of the enzymatic crosslinking led to a significant reduction of O_2_ and CO_2_ permeability, as well as to an increase in mechanical properties [6]. However, the blending of proteins with pectin polysaccharides in the presence of mTGase gave rise to films that exhibited enhanced mechanical properties in comparison with the blended films prepared without mTGase [7]. This effect was due to the fact that the microstructure of blended pectin/bitter vetch proteins in the presence of the enzyme was characterized by higher surface roughness and more homogeneous microstructure, with evident continuous zones respect to the blended films obtained without mTGase [7]. Finally, bitter vetch protein edible films nanostructured with mesoporous silica nanoparticles or with its amino-functionalized derivative and crosslinked by means of mTGase were obtained by Fernandez-Bats et al. [24]. The results indicated improved barrier properties when the nanocomposites were enzymatically crosslinked. In particular, in the presence of both the enzyme and amino-functionalized mesoporous silica, it was observed a reduction of almost 28%, 25%, and 70% of CO_2_, O_2,_ and H_2_O permeability, respectively. On the other hand, a worsening of about 50% of both tensile strength and elongation at break was observed in the presence of the enzyme. The results reported that the enzyme was able to improve both tensile strength and elongation at break. In addition, improved barrier properties toward water and gases like CO_2_ and H_2_O were observed when the nanocomposites were enzymatically crosslinked. As a matter of fact, morphology analyses demonstrated that nanoparticles were embedded more homogenously in the presence of mTGase [24]. 

#### 4.2.4. Grass Pea Proteins

Grass pea (*Lathyrus sativus* L.) is a very popular legume in many Asian and African countries where it is grown, especially for feeding animals and human consumption [97]. It is endowed with beneficial and useful biological as well as agronomic aspects, being resistant to dryness and possessing high grain-yielding capacity and high protein content. Grass pea flour is rich in molecular compounds with biological activity like phenolic substances having antioxidant properties. In addition, this flour is characterized by a low glycemic index [98]. In addition, its proteins, having the characteristic to be easily digested by the human gut, as demonstrated by in vitro gastric digestion carried out under physiological conditions [98,99], have glutamine and lysine residues able to be crosslinked by mTGase. It has been demonstrated that the mTGase-mediated treatment improved the nutritional properties of the flour [100]. Giosafatto et al. [25] used grass pea flour as a biopolymer source to prepare bioplastics after modifying the proteins by means of the microbial enzyme. The films cast by using the microbial enzyme-treated flour were 50% more transparent than the untreated ones. Moreover, the visualization by scanning electron microscopy demonstrated that the enzyme-modified films possessed a structure that was more compact and homogeneous. The enzyme also made films more resistant (tensile strength equal to 0.70 MPa for untreated films, and 1.40 MPa for the mTGase-treated ones), extensible (elongation at break equal to 59.1% for untreated films and 32.08% for the mTGase-treated ones) and less rigid (Young’s modulus equal to 26.2 MPa for untreated films and 17.1 for the mTGase-treated ones) than films prepared without the enzymatic tool. Finally, digestion experiments performed under physiological conditions indicated that the enzyme-treated coatings might lead the convey of bioactive molecules into the gastrointestinal tract, being the gastric and duodenal digestion rate lower than 50% and 32%, respectively, in the mTGase-crosslinked bioplastics [25]. 

## 5. Conclusions

Due to their intrinsic properties, proteins contained in by-products and wastes are suitable candidates for the manufacturing of bioplastics. Specifically, the protection of foods by means of edible films has been demonstrated not only to actively reduce the loss of moisture and flavors, but also to control gas exchange (oxygen, carbonic dioxide, and ethylene) and to allow the convey of biologically-active compounds (e.g., antimicrobials, antioxidants, or nutraceuticals). However, in terms of functional properties, bioplastics and edible films are still less efficient than synthetic polymer films. The use of mTGase has proven to be an effective and useful tool to improve different properties of protein-based bioplastics opening new insights into the production of biodegradable materials able to gradually replace the synthetic polymers. At present, there are various examples shown in this review in which mTGase-containing materials meet the requirements set by the industrial sector, although additional studies are needed for further improving the features of protein-based bioplastics.

## Figures and Tables

**Table 1:** Influence of mTGase-mediated modification on the technological features of different edible or bioplastic films obtained in the last two years (from 2018 to 2020).

Protein-Based Film Type	mTGase Effect on the Films	References
Bitter vetch proteins/mesoporous silica nanocomposite	Decrease in mechanical properties and decrease in gas permeability	[24]
Grass pea protein	Films more resistant and digested to a less extent under gastrointestinal physiological conditions	[25]
Coconut protein/guar gum	Enhancement of physico-chemical properties, such as mechanical, barrier properties and thermal features	[26]
Two quinoa varieties/chitosan	Enhancement of edible film physical properties	[27]
Collagen fiber/casein, keratin or SPI	Improved structure stability and packaging characters	[28]
Whey protein/heat ultrasounds	The properties of films were unaffected except their color	[29]
Whey protein concentrate/nanocrystalline cellulose	Mechanical properties enhancement	[30]
Whey protein isolate	Improved mechanical properties, gas permeability, and morphology properties	[31]
Quinoa protein/chitosan	Enhanced thermal stability and tensile strength. Elongation at break reduction	[32]
Nigella sativa seed proteins	Improved mechanical and barrier properties	[33]
Proteins from anchovy by-products	Improved mechanical, barrier, and surface properties	[34]

## Abbreviations

mTGase	microbial transglutaminase
MW	milk whey

## References

[1] Rajendran N., Puppala S., Sneha Raj M., Ruth Angeeleena B., Rajam C. (2012). Seaweeds can be a new source for bioplastics. J. Pharm. Res..

[2] Valdés A., Burgos N., Jiménez A., Garrigós M.C. (2015). Natural pectin polysaccharides as edible coatings. Coatings.

[3] Porta R. (2019). The plastics sunset and the bio-plastics sunrise. Coatings.

[4] Giosafatto C.V.L., Di Pierro P., Gunning P., Mackie A., Porta R., Mariniello L. (2014). Characterization of Citrus pectin edible films containing transglutaminase-modified phaseolin. Carbohydr. Polym..

[5] Ferreira M.S., Fai A.E.C., Andrade C.T., Picciani P.H., Azero E.G., Gonçalves É.C. (2016). Edible films and coatings based on biodegradable residues applied to acerolas (*Malpighia punicifolia* L.). J. Sci. Food Agric..

[6] Porta R., Di Pierro P., Rossi-Marquez G., Mariniello L., Kadivar M., Arabestani A. (2015). Microstructure and properties of bitter vetch (*Vicia ervilia)* protein films reinforced by microbial transglutaminase. Food Hydrocoll..

[7] Porta R., Di Pierro P., Sabbah M., Regalado-Gonzales C., Mariniello L., Kadivar M., Arabestani A. (2016). Blend films of pectin and bitter vetch (*Vicia ervilia*) proteins: Properties and effect of transglutaminase. Innov. Food Sci. Emerg. Technol..

[8] Al-Asmar A., Giosafatto C.V.L., Sabbah M., Sanchez A., Villalonga Santana R., Mariniello L. (2020). Effect of mesoporous silica nanoparticles on the physicochemical properties of pectin packaging material for strawberry wrapping. Nanomaterials.

[9] Geissdoerfer M., Savaget P., Bocken N.M.P., Hultin E.J. (2017). The circular economye A new sustainability paradigm?. J. Clean. Prod..

[10] Chen H., Wang J., Cheng Y., Wang C., Liu H., Bian H., Pan Y., Sun J., Han W. (2019). Application of protein-based films and coatings for food packaging: A Review. Polymers.

[11] Salama H.E., Aziz M.S.A., Sabaa M.W. (2018). Novel biodegradable and antibacterial edible films based on alginate and chitosan biguanidine hydrochloride. Int. J. Biol. Macromol..

[12] Popović S.Z., Lazić V.L., Hromiš N.M., Šuput D.Z., Bulut S.N., Grumezescu A.M., Holban A.M. (2018). Chapter 8—Biopolymer packaging materials for food shelf-life prolongation. Biopolymers for Food Design.

[13] Giosafatto C.V.L., Mariniello L., Ring S. (2007). Extraction and characterization of *Foeniculum vulgare* pectins and their use to prepare biopolymer films in the presence of phaseolin protein. J. Agric. Food Chem..

[14] Mariniello L., Giosafatto C.V.L., Moschetti G., Aponte M., Masi P., Sorrentino A., Porta R. (2007). Fennel waste-based films suitable for protecting cultivations. Biomacromolecules.

[15] Cuq B., Gontard N., Guilbert S. (1998). Proteins as agricultural polymers for packaging production. Cereal Chem..

[16] Pommet M., Redl A., Morel M.-H., Guilbert S. (2003). Study of wheat gluten plasticization with fatty acids. Polymer.

[17] Mariniello L., Di Pierro P., Esposito C., Sorrentino A., Masi P., Porta R. (2003). Preparation and mechanical properties of edible pectin-soy flour films obtained in the absence or presence of transglutaminase. J. Biotechnol..

[18] Mariniello L., Giosafatto C.V.L., Di Pierro P., Sorrentino A., Porta R. (2008). Use of the enzyme transglutaminase to prepare hydrocolloid-based edible films suitable for the food industry. J. Biotechnol..

[19] Mariniello L., Giosafatto C.V.L., Di Pierro P., Sorrentino A., Porta R. (2010). Swelling, mechanical, and barrier properties of albedo-based films prepared in the presence of phaseolin cross-linked or not by transglutaminase. Biomacromolecules.

[20] Porta R., Mariniello L., di Pierro P., Sorrentino A., Giosafatto C.V.L. (2011). Transglutaminase crosslinked pectin and chitosan-based edible films: A review. Crit. Rev. Food Sci. Nutr..

[21] Strop P. (2014). Versatility of microbial transglutaminase. Bioconjug. Chem..

[22] Giosafatto C.V.L., Al-Asmar A., Mariniello L., Kuddus M. (2018). Transglutaminase protein substrates of food interest. Enzymes in Food Technology: Improvement and Innovation.

[23] Sorrentino A., Giosafatto C.V.L., Sirangelo I., de Simone C., Di Pierro P., Porta R., Mariniello L. (2012). Higher susceptibility to amyloid fibril formation of the recombinant ovine prion protein modified by transglutaminase. Biochim. Biophys. Acta—Mol. Basis Dis..

[24] Fernandez-Bats I., Di Pierro P., Villalonga-Santana R., Garcia-Almendarez B., Porta R. (2018). Bioactive mesoporous silica nanocomposite films obtained from native and transglutaminase-crosslinked bitter vetch proteins. Food Hydrocoll..

[25] Giosafatto C.V.L., Al-Asmar A., D’Angelo A., Roviello V., Esposito M., Mariniello L. (2018). Preparation and characterization of bioplastics from grass pea flour cast in the presence of microbial transglutaminase. Coatings.

[26] Sorde K.L., Ananthanarayan L. (2019). Effect of transglutaminase treatment on properties of coconut protein-guar gum composite film. LWT—Food Sci. Technol..

[27] Escamilla-García M., Delgado-Sánchez L.F., Ríos-Romo R.A., García-Almendárez B.E., Calderón-Domínguez G., Méndez-Méndez J.V., Amaro-Reyes A., Di Pierro P., Regalado-González C. (2019). Effect of transglutaminase cross-linking in protein isolates from a mixture of two quinoa varieties with chitosan on the physicochemical properties of edible films. Coatings.

[28] Wu X., Luo Y., Liu Q., Jiang S., Mu G. (2019). Improved structure-stability and packaging characters of crosslinked collagen fiber-based film with casein, keratin and SPI. J. Sci. Food Agric..

[29] Cruz-Diaz K., Cobos Á., Fernández-Valle M.E., Díaz O., Cambero M.I. (2019). Characterization of edible films from whey proteins treated with heat, ultrasounds and/or transglutaminase. Application in cheese slices packaging. Food Packag. Shelf Life.

[30] Jiang S., Zhang T., Song Y., Qian F., Tuo Y., Mu G. (2019). Mechanical properties of whey protein concentrate based film improved by the coexistence of nanocrystalline cellulose and transglutaminase. Int. J. Biol. Macromol..

[31] Kouravand F., Jooyandeh H., Barzegar H., Hojjati M. (2020). mechanical, barrier and structural properties of whey protein isolate-based films treated by microbial transglutaminase. J. Microbiol. Biotech. Food Sci..

[32] Vera A., Tapia C., Abugoch L. (2020). Effect of high-intensity ultrasound treatment in combination with transglutaminase and nanoparticles on structural, mechanical, and physicochemical properties of quinoa proteins/chitosan edible films. Int. J. Biol. Macromol..

[33] Sabbah M., Altamimi M., Di Pierro P., Schiraldi C., Cammarota M., Porta R. (2020). Black edible films from protein-containing defatted cake of *Nigella sativa* seeds. Int. J. Mol. Sci..

[34] Yilmaz K., Turhan S., Saricaoglu F.T., Tural S. (2020). Improvement of physicochemical, mechanical, thermal and surface properties of anchovy by-product protein films by addition of transglutaminase, and the correlation between secondary structure and mechanical properties. Food Packag. Shelf Life.

[35] Shoulders M.D., Raines R.T. (2009). Collagen structure and stability. Annu. Rev. Biochem..

[36] Cheng S., Wang W., Li Y., Gao G., Zhang K., Zhou J., Wu Z. (2019). Cross-linking and film-forming properties of transglutaminase-modified collagen fibers tailored by denaturation temperature. Food Chem..

[37] Wu X., Liu A., Wang W., Ye R. (2018). Improved mechanical properties and thermal-stability of collagen fiber based film by crosslinking with casein, keratin or SPI: Effect of crosslinking process and concentrations of proteins. Int. J. Biol. Macromol..

[38] Wu X., Liu Y., Liu A., Wang W. (2017). Improved thermal-stability and mechanical properties of type I collagen by crosslinking with casein, keratin and soy protein isolate using transglutaminase. Int. J. Biol. Macromol..

[39] Park S.Y., Lee B.I., Jung S.T., Park H.J. (2001). Biopolymer composite films based on κ-carrageenan and chitosan. Mater. Res. Bull..

[40] Kim J.S., Park J.W. (2005). Partially purified collagen from refiner discharge of pacific whiting surimi processing. J. Food Sci..

[41] Yi J.B., Kim Y.T., Bae H.J., Whiteside W.S., Park H.J. (2006). Influence of transglutaminase-induced cross-linking on properties of fish gelatin films. J. Food Sci..

[42] Alvarado S., Sandoval G., Palos I., Tellez S., Aguirre-Loredo Y., Velazquez G. (2015). The effect of relative humidity on tensile strength and water vapor permeability in chitosan, fish gelatin and transglutaminase edible films. Food Sci. Technol..

[43] Au Y. (2004). The muscle ultrastructure: A structural perspective of the sarcomere. Cell. Mol. Life Sci..

[44] Chen X., Zou Y., Han M., Pan L., Xing T., Xu X., Zhou G. (2016). Solubilization of myosin in a solution of low ionic strength L-histidine: Significance of the imidazole ring. Food Chem..

[45] Shiku Y., Hamaguchi P.Y., Tanaka M. (2003). Effect of pH on the preparation of edible films based on fish myofibrillar proteins. Fish. Sci..

[46] Cercel F., Stroiu M., Alexe P., IaniĠchi D. (2015). Characterization of myofibrillar chicken breast proteins for obtain protein films and biodegradable coating generation. Agric. Agric. Sci. Proced..

[47] Yayli D., Turhan S., Saricaoglu F.T. (2017). Edible packaging film derived from mechanically deboned chicken meat proteins: Effect of transglutaminase on physicochemical properties. Korean J. Food Sci. Anim. Resour..

[48] Kaewprachu P., Osako K., Tongdeesoontorn W., Rawdkuen S. (2017). The effects of microbial transglutaminase on the properties of fish myofibrillar protein film. Food Packag. Shelf Life.

[49] Rostamzad H., Paighambari S.Y., Shabanpour B., Ojagh S.M., Mousavi S.M. (2016). Improvement of fish protein film with nanoclay and transglutaminase for food packaging. Food Packag. Shelf Life.

[50] Dalgleish D.G., Corredig M. (2012). The structure of the casein micelle of milk and its changes during processing. Annu. Rev. Food Sci. Technol..

[51] Birgitte M.C., Esben S.S., Peter H., Torben E.P., Lone K.R. (1996). Localization of potential transglutaminase cross-linking sites in bovine caseins. J. Agric. Food Chem..

[52] Chambi H., Grosso C. (2006). Edible films produced with gelatin and casein cross-linked with transglutaminase. Food Res. Int..

[53] Sohail S.S., Wang B., Biswas M.A.S., Oh J.-H. (2006). Physical, morphological, and barrier properties of edible casein films with wax applications. J. Food Sci..

[54] Peng N., Gu L., Li J., Chang C., Li X., Su Y., Yang Y. (2017). Films based on egg white protein and succinylated casein cross-linked with transglutaminase. Food Bioprocess Technol..

[55] Yadav J.S.S., Yan S., Pilli S., Kumar L., Tyagi R.D., Surampalli R.Y. (2015). Cheese whey: A potential resource to transform into bioprotein, functional/nutritional proteins and bioactive peptides. Biotechnol. Adv..

[56] Abdalrazeq M., Giosafatto C.V.L., Esposito M., Fenderico M., Di Pierro P., Porta R. (2019). Glycerol-Plasticized films obtained from whey proteins denatured at alkaline pH. Coatings.

[57] Limsawat P., Pruksasri S. (2010). Separation of lactose from milk by ultrafiltration. Asian J. Food Agro-Ind..

[58] Korhonen H. (2009). Milk-derived bioactive peptides: From science to applications. J. Funct. Foods..

[59] Tarhan O., Spotti M.J., Schaffter S., Corvalan C.M., Campanella O.H. (2016). Rheological and structural characterization of whey protein gelation induced by enzymatic hydrolysis. Food Hydrocoll..

[60] Di Pierro P., Rossi-Marquez G., Mariniello L., Sorrentino A., Villalonga R., Porta R. (2013). Effect of transglutaminase on the mechanical and barrier properties of whey protein/pectin films prepared at complexation pH. J. Agric. Food Chem..

[61] Di Pierro P., Chico B., Villalonga R., Mariniello L., Damiao A.E., Masi P., Porta R. (2006). Chitosan-whey protein edible films produced in the absence or presence of transglutaminase: Analysis of their mechanical and barrier properties. Biomacromolecules.

[62] Hernàndez-Balada E., Taylor M.M., Phillips J.G., Marmer W.N., Brown E.M. (2009). Properties of biopolymers produced by transglutaminase treatment of whey protein isolate and gelatin. Bioresour. Technol..

[63] Bernhisel-Broadbent J., Dintzis H.M., Dintzis R.Z., Sampson H.A. (1994). Allergenicity and antigenicity of chicken egg ovomucoid (Gal d III) compared with ovalbumin (Gal d I) in children with egg allergy and in mice. J. Allergy. Clin. Immunol..

[64] Langeland T. (1982). A clinical and immunological study of allergy to hen’s egg white. II. Antigens in hen’s egg white studied by crossed immunoelectrophoresis (CIE). Allergy.

[65] Lim L.-T., Mine Y., Tung M.A. (1998). Transglutaminase cross-linked egg white protein films:  tensile properties and oxygen permeability. J. Agric. Food Chem..

[66] Giosafatto C.V.L., Rigby N.M., Wellner N., Ridout M., Husband F., Mackie A.R. (2012). Microbial transglutaminase-mediated modification of ovalbumin. Food Hydrocoll..

[67] Di Pierro P., Chico B., Villalonga R., Mariniello L., Masi P., Porta R. (2007). Transglutaminase-catalyzed preparation of chitosan-ovalbumin films. Enzyme Microb. Technol..

[68] Marcet I., Sáez S., Rendueles M., Díaz M. (2017). Edible films from residual delipidated egg yolk proteins. J. Food Sci. Technol..

[69] Gao Y., Janes M.E., Chaiya B., Brennan M.A., Brennan C.S., Prinyawiwatkul W. (2017). Gluten-free bakery and pasta products: Prevalence and quality improvement. Int. J. Food Sci. Technol..

[70] Schoenlechner R., Mandala I., Kiskini A., Kostaropoulos A., Berghofer E. (2010). Effect of water, albumen and fat on the quality of gluten-free bread containing amaranth. Int. J. Food Sci. Technol..

[71] Romano A., Masi P., Bracciale A., Aiello A., Nicolai M.A., Ferranti P. (2018). Effect of added enzymes and quinoa flour on dough characteristics and sensory quality of a gluten-free bakery product. Eur. Food Res. Technol..

[72] Coltelli M.-B., Wild F., Bugnicourt E., Cinelli P., Lindner M., Schmid M., Weckel V., Müller K., Rodriguez P., Staebler A. (2016). State of the art in the development and properties of protein-based films and coatings and their applicability to cellulose based products: An extensive review. Coatings.

[73] Jinshui W., Yuwei Z., Mouming Z. (2005). Development and physical properties of film of wheat gluten cross-linked by transglutaminase. J. Wuhan Univ. Technol..

[74] Larré C., Desserme J., Barbot J., Gueguen J. (2000). Properties of deamidated gluten films enzymatically crosslinked. J. Agric. Food Chem..

[75] Lai H., Chiang I.-C. (2006). Properties of MTGase treated gluten film. Eur. Food Res. Technol..

[76] Cui L., Yuan J., Wang P., Sun H., Fan X., Wang Q. (2017). Facilitation of α-polylysine in TGase-mediated cross- linking modification for gluten and its effect on properties of gluten films. J. Cereal Sci..

[77] Landry J. (1997). Comparison of extraction methods for evaluating zein content of maize grain. Cereal Chem..

[78] Dickey L.C., Parris N. (2002). Serial batch extraction of zein milled maize. Ind. Crops Prod. Int. J..

[79] Cuq B., Gontard N., Guilbert S., Rooney M.L. (1995). Edible films and coatings as active layers. Active Food Packaging.

[80] Wang Y., Padua G.W. (2006). Water barrier properties of zein-oleic acid films. Cereal Chem..

[81] Barbosa de Almeida C., Tafari Catelam K., Lopes Cornélio M., Lopes Filho J. (2010). Morphological and structural characteristics of zein biofilms with added xanthan gum. Food Technol. Biotechnol..

[82] Pena Serna C., Lopes Filho J.F. (2015). Biodegradable Zein-Based Blend Films: Structural, Mechanical and Barrier Properties. Food Technol. Biotechnol..

[83] Oh J.H., Wang B., Field P.D., Aglan H.A. (2004). Characteristics of edible films made from dairy proteins and zein hydrolysate cross-linked with transglutaminase. Int. J. Food Sci. Technol..

[84] Masamba K., Li Y., Hategekimana J., Liu F., Ma J., Zhong F. (2016). Effect of type of plasticizers on mechanical and water barrier properties of transglutaminase cross-linked zein-oleic acid composite films. Int. J. Food Eng..

[85] Masamba K., Li Y., Hategekimana J., Zehadi M., Ma J., Zhong F. (2016). Evaluation of mechanical and water barrier properties of transglutaminase cross-linked zein films incorporated with oleic acid. Int. J. Food Sci. Technol..

[86] Masamba K., Li Y., Hategekimana J., Ma J., Zhong F. (2016). Effect of drying temperature and pH alteration on mechanical and water barrier properties of transglutaminase cross linked zein-oleic acid composite films. LWT—Food Sci. Technol..

[87] Xing G., Giosafatto C.V.L., Xin R., Dong M., Mariniello L. (2019). Microbial transglutaminase-mediated polymerization in the presence of lactic acid bacteria affects antigenicity of soy protein component present in bio-tofu. J. Funct. Foods.

[88] Xing G., Giosafatto C.V.L., Carpentieri A., Pasquino R., Dong M., Mariniello L. (2020). Gelling behavior of bio-tofu coagulated by microbial transglutaminase combined with lactic acid bacteria. Food Res. Int..

[89] Di Pierro P., Mariniello L., Giosafatto C.V.L., Masi P., Porta R. (2005). Solubility and permeability properties of edible pectin-soy flour films obtained in the absence or presence of transglutaminase. Food Biotechnol..

[90] Di Pierro P., Mariniello L., Sorrentino A., Villalonga R., Chico B., Porta R. (2010). Putrescine-polysaccharide conjugates as transglutaminase substrates and their possible use in producing cross-linked films. Amino Acids.

[91] Tang C.H., Jiang Y., Wen Q.-B., Yang X.-Q. (2005). Effect of transglutaminase treatment on the properties of cast films of soy protein isolates. J. Biotechnol..

[92] Jiang Y., Wen Q.-B., Tang C.-H., Yang X.-Q. (2006). Effect of transglutaminase on properties of cast films from food proteins. J. South. Chin. Univ. Technol. (Nat. Sci.).

[93] Romano A., Giosafatto C.V.L., Di Pierro P., Romano R., Masi P., Mariniello L. (2016). Impact of transglutaminase treatment on properties and in vitro digestibility of white bean (*Phaseolus vulgaris* L.) flour. Food Res. Int..

[94] Romano A., Giosafatto C.V.L., Masi P., Mariniello L. (2015). Impact of dehulling on the physico-chemical properties and in vitro protein digestion of common beans (*Phaseolus vulgaris* L.). Food Funct..

[95] Arabestani A., Kadivar M., Amoresano A., Illiano A., Di Pierro P., Porta R. (2016). Bitter vetch (*Vicia ervilia*) seed protein concentrate as possible source for production of bilayered films and biodegradable containers. Food Hydrocoll..

[96] Sabbah M., Di Pierro P., Giosafatto C.V.L., Esposito M., Mariniello L., Regalado-Gonzales C., Porta R. (2017). Plasticizing Effects of Polyamines in Protein-Based Films. Int. J. Mol. Sci..

[97] Granati E., Bisignano V., Chiaretti D., Crino P., Polignano G.B. (2003). Characterization of Italian and exotic Lathyrus germplasm for quality traits. Genet. Resour. Crop. Evol..

[98] Romano A., Giosafatto C.V.L., Al-Asmar A., Masi P., Aponte M., Mariniello L. (2019). Grass pea (*Lathyrus sativus*) flour: Microstructure, physico-chemical properties and in vitro digestion. Eur. Food Res. Technol..

[99] Shani-Levi C., Alvito P., Andrés A., Assunço R., Barbera R., Blanquet-Diot S., Bourlieug C., Brodkorbi A., Cillae A., Deglaireh A. (2017). Extending in vitro digestion models to specific human populations: Expert perspectives, practical tools and bio-relevant information. Trends Food Sci. Technol..

[100] Romano A., Giosafatto C.V.L., Al-Asmar A., Masi P., Romano R., Mariniello L. (2019). Structure and in vitro digestibility of grass pea (*Lathyrus sativus* L.) flour following transglutaminase treatment. Eur. Food Res. Technol..

